# Virtual Reality and Sensing Techniques for Humans

**DOI:** 10.3390/s26216979

**Published:** 2026-11-04

**Authors:** Răzvan Gabriel Boboc, Ali Vatankhah Barenji

**Affiliations:** 1 Department of Automotive and Transport Engineering, Transilvania University of Brașov, 500036 Brasov, Romania; 2 Milton Stewart School of Industrial and Systems Engineering, Georgia Institute of Technology, Atlanta, GA 30332-0205, USA; ali.barenji@nmt.edu

## 1. Introduction

Over the past decade, virtual reality (VR) has evolved from a niche entertainment medium into a mature and versatile technological ecosystem supporting scientific research, education, healthcare, industrial training, human performance analysis, and interactive system design [1,2,3]. Advances in display technologies, real-time rendering, and affordable head-mounted displays (HMDs) have significantly lowered barriers to adoption, enabling immersive environments that can be systematically controlled, replicated, and instrumented for experimental and applied purposes [4].

A defining characteristic of contemporary VR systems is their increasingly tight integration with sensing technologies. Modern VR platforms routinely incorporate motion tracking, inertial measurement units, depth cameras, eye-tracking systems, physiological sensors, and haptic interfaces, allowing for the continuous, high-resolution measurement of user behavior and internal states [5,6]. These sensing modalities transform VR from a purely visual experience into a powerful human-centered measurement platform capable of capturing kinematic, cognitive, emotional, and physiological responses in real time.

The convergence of VR and sensing technologies has proven particularly valuable for studying human behavior under conditions that are difficult, unsafe, or impractical to reproduce in the real world. Applications range from clinical rehabilitation and mental health assessment to ergonomic evaluation, driving simulation, collaborative work analysis, and stress or fatigue monitoring [7,8,9]. By embedding sensors within immersive environments, researchers can observe naturalistic user responses while maintaining experimental control, thereby improving ecological validity without sacrificing measurement precision.

Recent research has also emphasized the importance of multimodal sensing and data fusion within VR environments. Combining heterogeneous data streams, such as gaze behavior, body posture, biosignals, and interaction logs, enables richer models of user intent, workload, and engagement [10]. Machine learning and deep learning techniques play an increasingly central role in this process, facilitating real-time classification, prediction, and adaptation based on complex sensor data [11]. These algorithmic advances support the development of intelligent VR systems that respond dynamically to user states, enabling personalized, adaptive, and context-aware immersive experiences.

Furthermore, there is also a growing emphasis on accessibility and scalability. The emergence of low-cost sensors and markerless tracking solutions has expanded the reach of VR-based sensing beyond specialized laboratories into classrooms, clinics, and industrial environments [12,13,14]. This democratization of technology encourages broader participation and accelerates innovation across disciplines.

In this context, the present Special Issue of *Sensors*, entitled “Virtual Reality and Sensing Techniques for Humans”, was conceived to bring together cutting-edge research that explores the methodological, technological, and application-oriented dimensions of sensor-integrated VR systems. By highlighting advances in sensing modalities, data analysis techniques, and human-centered applications, this Special Issue aims to provide a comprehensive snapshot of current progress while identifying emerging challenges and future research directions at the intersection of immersive technologies and human sensing.

## 2. An Overview of the Special Issue’s Contributions

This Special Issue brings together ten contributions (nine original research articles and one comprehensive review) that explore diverse aspects of virtual reality (VR) and sensing techniques, spanning behavioral analysis, interaction methods, machine learning approaches, ergonomic assessment, and sensor system design.

### 2.1. Biomechanical Risk Assessment via Markerless Motion Capture

Gennarelli et al. compare wearable sensor networks with markerless motion capture systems for ergonomic analysis of lifting tasks, focusing on biomechanical risk indices (Contribution 1). Their findings suggest that machine-learning-enhanced markerless systems can produce reliable kinematic estimates and, when coupled with wearable data, support more accurate and repeatable assessments in occupational settings.

### 2.2. Virtual Reality as a Stress Measurement Platform

Rah and Chen present a novel framework that leverages VR as a primary platform for real-time behavioral analysis, combining minimal physiological sensing (galvanic skin response) with VR-based behavioral cues to assess stress responses (Contribution 2). Their Sensor-Assisted Unity Architecture demonstrates how behavioral data from VR environments, enhanced via lightweight physiological input, can improve detection sensitivity without relying on complex multi-sensor systems.

### 2.3. Enhancing Design Education with Low-Cost Eye Tracking

Rojas, Higuera-Trujillo, and Vergara investigate the integration of low-cost eye-tracking devices and alternative tracking methods into a product design course (Contribution 3). Through surveys with design students, they show that both technologies, especially eye tracking, are perceived as useful, novel, and relevant, underscoring the potential for affordable sensing tools to enrich design pedagogy and student engagement.

### 2.4. User Experience of Virtual Human Role-Playing in Psychological Assessment

Magar, Suk, and Laine explore user perceptions of immersive role-playing with virtual humans (VHs) within a psychological testing tool called EmpathyVR (Contribution 4). By examining immersion, embodiment, and satisfaction among adolescent participants, this study highlights the promise and challenges of using VR role-playing scenarios for psychological assessment, particularly in empathy-related contexts.

### 2.5. Deep Learning for Mental Fatigue Assessment

Fan and colleagues introduce a hybrid deep learning model that utilizes a combination of ECG signal inputs, including raw time series data, spectral representations, and physiological features, to classify mental fatigue states (Contribution 5). Their approach, integrating ResNet and Bi-LSTM with transformer-based fusion, achieves high classification accuracy and demonstrates the potential for advanced neural architectures to assess cognitive states from physiological sensors.

### 2.6. Intrusion Detection Through Hybrid Petri Net and LSTM Models

Volpe and co-authors propose a hybrid intrusion detection system that integrates Long Short-Term Memory (LSTM) neural networks with Petri Net models for the real-time identification and mitigation of malicious network traffic (Contribution 6). Although primarily focused on cybersecurity, the work emphasizes virtual sensing technologies in complex cyber–physical and virtual environments, illustrating how hybrid models can enhance detection accuracy and operational adaptability.

### 2.7. Wearable Haptic Displays with Optical Calibration

Frediani and Carpi present an optical calibration technique for wearable tactile displays that render softness cues in VR (Contribution 7). By monitoring the skin contact area with an external camera and generating calibration curves, they achieve controlled tactile feedback, a key step toward richer haptic rendering in immersive environments.

### 2.8. Head Movement Classification in Immersive VR

Callahan-Flintoft and colleagues evaluate multiple algorithms for classifying head movements based on data streams from head-mounted displays (Contribution 8). By comparing algorithmic outputs with human rater annotations, they provide practical recommendations for researchers for selecting and implementing head movement classification methods in VR studies.

### 2.9. Analysis of Electronic Nose Data with Machine Learning

Chang, Byun, Lim, and Park investigate electronic nose (e-nose) sensor data using deep learning and boosting approaches to evaluate learning efficiency and accuracy (Contribution 9). Their results indicate that boosting models excel on structured data, outperforming several deep learning configurations and showcasing effective machine learning strategies for chemical sensing contexts relevant to VR applications, such as environmental perception or safety monitoring.

### 2.10. Review—Wearable Sensors in VR Driving Simulators

The review by Boboc, Butilă, and Butnariu synthesizes the literature on the use of wearable sensors in VR driving simulators for driver behavior and state assessment (Contribution 10). Covering 44 studies over a decade, this review highlights sensor types, experimental setups, and methodological trends, providing a valuable overview of how wearable technologies complement VR for immersive, controlled evaluations in transportation and human performance research.

## 3. Emerging Trends and Future Directions

The contributions in this Special Issue highlight several key trends shaping the evolution of virtual reality (VR) and sensing technologies for human-centered applications. Rather than progressing along a single technological path, the field is advancing through the convergence of multimodal sensing, computational intelligence, and immersive interaction design, enabling increasingly responsive and adaptive virtual environments.

A prominent trend is the growing emphasis on multimodal sensing integration for a comprehensive assessment of the human state. The studies demonstrate that single sensing modalities are often insufficient to capture the complexity of human behavior. Instead, combining motion data from VR tracking systems with physiological signals, eye tracking, and interaction logs enables richer representations of cognitive workload, emotional response, fatigue, stress, and engagement. Future research will need to focus on robust strategies for data fusion, synchronization, and uncertainty management across heterogeneous sensing streams.

Another important direction concerns the democratization and scalability of VR-based sensing. Several contributions show that meaningful insights can be obtained using low-cost sensors, lightweight physiological measurements, and markerless tracking when embedded in well-designed virtual environments. This shift reflects a move away from highly specialized laboratory setups toward accessible systems suitable for education, healthcare, and industrial applications. A key challenge moving forward will be balancing accessibility with measurement validity and reliability across diverse contexts and populations.

The increasing role of machine learning and artificial intelligence represents a third defining trend. Advanced models are now routinely applied to interpret the complex, high-dimensional data generated in immersive environments. As these methods mature, future work is expected to transition from offline analysis to real-time inference and closed-loop adaptation, enabling VR systems to dynamically adjust task demands, feedback, or environmental conditions in response to user state.

An emerging paradigm is the move toward human–system symbiosis, in which sensing, interpretation, and adaptation occur continuously. This approach supports intelligent VR environments that respond to user needs and intentions, fostering more natural and supportive interactions. Achieving this vision will require advances in sensing, algorithms, interaction design, and user trust.

Despite these advances, challenges related to data standardization, cross-platform comparability, long-term reliability, and ethical considerations—such as privacy and psychological impacts—remain. Addressing these issues will require interdisciplinary collaboration and the development of shared methodological frameworks.

Overall, the trends identified in this Special Issue point toward integrated, intelligent, and human-centered VR sensing ecosystems. Continued progress will depend on combining methodological rigor with technological innovation to fully realize VR’s potential as both an immersive medium and a powerful tool for understanding and supporting human behavior.

## 4. Conclusions

The contributions presented in this Special Issue highlight the growing maturity and interdisciplinary character of sensor-integrated virtual reality (VR) systems for human-centered research and applications. Collectively, the articles demonstrate how advances in sensing technologies, data analysis methods, and immersive platforms are enabling more precise, flexible, and ecologically valid investigations of human behavior, cognition, and performance within controlled yet realistic virtual environments.

Beyond serving as a medium for visualization or interaction, VR is increasingly emerging as a comprehensive experimental and analytical ecosystem. By embedding sensing, data fusion, and intelligent processing directly into immersive environments, VR platforms can function as adaptive “living laboratories” that support repeatable experimentation, longitudinal assessment, and ethically controlled exposure to complex or safety-critical scenarios. This capability is particularly valuable for applications in rehabilitation, ergonomics, training, psychological assessment, and human factor research.

Looking forward, continued progress in this field will depend on the improved standardization, validation, and interoperability of VR sensing methodologies, as well as careful attention to ethical considerations related to data privacy, bias, and user well-being. Addressing these challenges will require sustained interdisciplinary collaboration across engineering, computer science, psychology, design, and applied domains.

In summary, this Special Issue provides a timely overview of current advances at the intersection of virtual reality and sensing technologies. By showcasing diverse methods, applications, and emerging research directions, it aims to stimulate further innovation and collaboration toward the development of robust, intelligent, and human-centered VR sensing systems with broad scientific and societal impact.
